# A Review of mTOR Pathway Inhibitors in Gynecologic Cancer

**DOI:** 10.1155/2017/4809751

**Published:** 2017-02-13

**Authors:** Andréia Cristina de Melo, Eduardo Paulino, Álvaro Henrique Ingles Garces

**Affiliations:** Brazilian National Cancer Institute (INCA), Rio de Janeiro, RJ, Brazil

## Abstract

The treatment of advanced gynecologic cancers remains palliative in most of cases. Although systemic treatment has entered into the era of targeted drugs the antitumor efficacies of current therapies are still limited. In this context there is a great need for more active treatment and rationally designed targeted therapies. The PI3K/AKT/mTOR is a signaling pathway in mammal cells that coordinates important cell activities. It has a critical function in the survival, growth, and proliferation of malignant cells and was object of important research in the last two decades. The mTOR pathway emerges as an attractive therapeutic target in cancer because it serves as a convergence point for many growth stimuli and, through its downstream substrates, controls cellular processes that contribute to the initiation and maintenance of cancer. Aberrant PI3K-dependent signaling occurs frequently in a wide range of tumor types, including endometrial, cervical, and ovarian cancers. The present study reviewed the available evidence regarding the potential impact of some mTOR pathway inhibitors in the treatment of gynecological cancer. Few advances in medical management have occurred in recent years in the treatment of advanced or recurrent gynecological malignancies, and a poor prognosis remains. Rationally designed molecularly targeted therapy is an emerging and important option in this setting; then more investigation in PI3K/AKT/mTOR pathway-targeted therapies is warranted.

## 1. Introduction

The treatment of advanced gynecologic cancers remains palliative in most of cases and the vast majority of the patients will eventually die. Although systemic treatment has entered into the era of targeted drugs the antitumor efficacies of current therapies are still limited, most likely because of the high degree of cancer clonal heterogeneity and cell signal complexity [1]. In this context there is a great need for more active treatment and rationally designed targeted therapies [2].

The PI3K/AKT/mTOR is a signaling pathway in mammal cells that coordinates important cell activities [2]. It has a critical function in the survival, growth, and proliferation of malignant cells and was object of important research in the last two decades [3–5]. The deregulation of the mammalian target of rapamycin (mTOR) and other proteins of this pathway occurs in many solid tumors and tumor cells have more sensitivity to mTOR inhibitors than normal cells [6]. Mechanisms for pathway activation include loss of tumor suppressor* PTEN* (phosphatase and tensin homolog) function, amplification or mutation of* PI3K* (phosphoinositide 3-kinase), amplification or mutation of* AKT* (protein kinase B), activation of growth factor receptors, and exposure to carcinogens [7, 8].

The mTOR pathway emerges as an attractive therapeutic target in cancer because it serves as a convergence point for many growth stimuli and, through its downstream substrates, controls cellular processes that contribute to the initiation and maintenance of cancer [8]. Aberrant PI3K-dependent signaling occurs frequently in a wide range of tumor types, including endometrial, cervical, and ovarian cancers [2, 9].

## 2. Endometrial Cancer

Endometrial cancer (EC) is the most common and the second cause of death among gynecologic cancers in United States, with more than 60.000 new cases and 10.000 deaths expected in 2016 [10]. Unfortunately, data from 2013 [11] shows that EC research received far less funding than ovarian cancer ($17.8 versus $100.8 million, resp.) and this uneven funding translates in almost four times less research projects for EC compared to ovarian cancer (488 versus 1785, resp.) [12].

Initial approach to EC is surgical staging with hysterectomy plus salpingoforectomy, with or without lymph node assessment. Adjuvant treatment is based on risk factors (FIGO stage, histology, grade, etc.) and nowadays patients are receiving more systemic treatment upfront, even in early stage disease [13, 14]. For those with advanced and recurrent disease, treatment options are much more limited, with a doublet of platinum salt and taxane for first-line treatment and no standard approach for future lines of therapy.

Historically, EC was divided into type I (mainly endometrioid histology) and type II (nonendometrioid) carcinomas but this classification does not take into account the molecular profiles of tumors [15]. In the last decade more attention has been given to molecular pathways and like many other types of cancers target therapy emerged as an excellent option of treatment. In TCGA project for EC [16] (mainly endometrioid and serous histology) four molecular subgroups of EC were seen: POLE-ultramutated, MSI-hypermutated, copy number high (serous-like), and copy number low, with each subgroup showing different altered molecular pathways.

PI3K/AKT/mTOR is the most important altered pathway in EC and it seems to harbor the highest alterations among all solid tumors. Oza et al. [17] reported that this pathway could be target with mTOR inhibitor (temsirolimus) and it became one of the milestones in EC. Since that many trials were published targeting PI3K/AKT/mTOR pathway with promising results.

### 2.1. PI3K/AKT/mTOR Pathway and Endometrial Cancer

The TCGA reported in 2013 the molecular profile of EC [16]. It collected data from 373 patients with endometrioid and serous adenocarcinoma (clear cell was not represented). A genomic, transcriptomic, and proteomic analysis was done using array and sequencing-based technologies and four major molecular profiles were found: POLE (ultramutated), MSI (hypermutated), copy number low (CNL, endometrioid), and copy number high (CNH, serous-like), with distinct outcomes in progression-free survival (PFS) (POLE and CNH subgroups have the highest and poorest outcome, resp., [*p* = 0.02]). In general, EC has alterations on* PI3KCA*,* PIK3R1*,* AKT1,* and* PTEN* in about 59.7%, 33%, 3.2%, and 66% of cases, respectively. When subgroups are analyzed separated, POLE has the highest rate of* PTEN*,* PI3KCA,* and* PIK3R1* alterations: 94%, 71%, and 65%, respectively. On the other side, CNH has different type of mutations/amplifications, with* P53* being the most altered in 92% of the EC (*PI3K* 47% and* PTEN* 11%). Also, CNH showed HER2 amplifications in 25% of EC.

### 2.2. Therapeutics with PI3K/AKT/mTOR Inhibitors

There are many drugs being tested in each part of the pathway: inhibiting PI3K, mTOR, AKT, and dual inhibitors on PI3K/mTOR and PI3K/AKT. Until now the most tested drugs are those blocking mTOR activity. Oza et al. [17] showed promising results with temsirolimus in 54 recurrent or metastatic EC patients with no (*n* = 29) or prior (*n* = 25) lines of therapy. Chemotherapy-naïve patients had 14% of response rate (RR), comparing to 4% on chemotherapy-treated group. A recent randomized phase 2 study compared single-agent ridaforolimus to progestins or investigator's choice chemotherapy in 130 patients with advanced EC and prior systemic treatments [18]. Patients in ridaforolimus arm had higher PFS (3.6 × 1.9 months, *p* = 0.008) and more stable disease (SD) (35% versus 27%, *p* = 0.021), but also more grade 3/4 adverse events like diarrhea (11.1% versus 1.5%), hyperglycemia (19% versus 0%), and anemia (12.7% versus 4.6%). Many other trials have been testing mTOR inhibitors to date and taking together these trials showed moderate activity with overall response rate (ORR) varying from 4 to 24% (with higher responses for those chemotherapy-naïve) and prolonged SD [19–23]. Toxicity profile was manageable with the most reported events being hematological, metabolically, constitutional, and gastrointestinal. PI3K and AKT inhibitors are also being studied. Pilaralisib [24], an PI3K inhibitor, showed minimal efficacy in recurrent EC patients who received prior systemic therapy, with RR of 6% and SD of 37.3%, and the AKT inhibitor MK-2206 [25] showed minimal activity with 5.5% RR and 33% SD in 36 patients with prior systemic therapies for recurrent EC with high rate of grade 3/4 adverse events (58%).

Emerging strategies are trying to combine drugs that have different ways of action in order to overcome resistance and some studies have shown the feasibility of this strategy. On a phase 2 trial [26], patients were randomized between temsirolimus with or without megestrol acetate alternating with tamoxifen. This study reported no improvement on efficacy and unfortunately closed early due to excess of venous thrombosis. Promising clinical activity was seen combining everolimus plus anastrozole. Slomovitz et al. [27] showed an ORR of 32% in patients with prior systemic therapies who were treated with this combination, and the responses were even higher in those women who were taking metformin for diabetes (ORR of 55%). Based on these astonishing results with the triplet regimen, the authors planned another phase II trial and presented the results at ASCO 2016 [28]. Patients treated with the combination of everolimus, anastrozole, and metformin had an ORR of 29%, with 60% having clinical benefit rate [complete response (CR) + partial response (PR) + SD]. Antiangiogenic agents also showed to be active on EC (mainly bevacizumab) and studies combining these drugs with mTOR inhibitors were designed and tested. Alvarez et al. [29] reported that in pretreated EC this combination demonstrated an ORR of 25%, but with high rate of toxicities (39% of patients discontinued treatment for toxicity). Einstein et al. [30] reported the results of this combination on pretreated EC and unfortunately this study did not meet the prespecified efficacy criteria. They reported PR and SD in 20% and 48% of patients at 6 months, respectively.

Recently, Aghajanian et al. [31] presented a randomized phase II trial, comparing carboplatin and paclitaxel with either temsirolimus or bevacizumab or carboplatin plus ixabepilone and bevacizumab to the historical control arm of GOG 209 (carboplatin and paclitaxel arm). This study showed improved overall survival (OS) for the antiangiogenic therapy when added to carboplatin and paclitaxel backbone, but unfortunately temsirolimus did not improve outcomes compared to historical control arm.

Based on some studies suggesting that blocking the PI3K/AKT/mTOR pathway could interfere with and cause defects in the DNA repair mechanism [32], like homologous recombination, much interest has emerged combining these pathway inhibitors with PARP inhibitors [33]. On cells with DNA double-strand breaks, PTEN loss may dysfunct the homologous recombination repair, “mimicking” BRCA1/2 mutation. Combining PI3K/AKT/mTOR and PARP inhibitors could recapitulate the synthetic lethality observed in ovarian cancer patients with BRCA germ line mutation treated with olaparib. Gathering the high rate of PI3K alterations and PTEN loss in EC, it seems this is a promising strategy in this neoplasia. One recent case [34] showed the activity of olaparib in a patient with metastatic endometrial cancer, BRCA negative and heavily pretreated with multiple lines of platinum compound, taxane and doxorubicin. This patient showed PR in liver, lung, and brain lesions.

Recent progress on immune mediators in oncology brought a lot of hope of better and more efficient drugs to treat cancer. And this fact is not different for gynecologic cancers. Some evidences emerged showing the importance of mTOR in optimizing the immune response. It has a role in antigen presentation by dendritic cells as well as promoting expression of CD86 (stimulatory molecules) and decreasing PDL1 on T cells (inhibitory molecules). Based on this fact, some phase I studies are evaluating the role of mTOR inhibitors with therapeutic vaccines (NCT01522820).

## 3. Cervical Cancer

Cervical cancer (CC) is a public health problem, representing the fourth most commonly diagnosed cancer and the seventh overall, with an estimated 528,000 new cases in 2012 across the world [35]. There were an estimated 266,000 deaths from CC worldwide in 2012, accounting for 7.5% of all female cancer deaths and this number is expected to increase up to 410,000 by 2030 [36]. Each year, approximately 200,000 women die of this disease. Developing countries account for approximately 76 to 85% of CC cases [36].

Virtually all CCs (more than 99%) are caused by high-risk human papillomavirus (HPV) [37]. The HPV E7 oncoprotein is essential for CC carcinogenesis. The AKT phosphorylation demonstrated in samples of CC suggests a constitutive activation of the PI3K/AKT pathway in patients [38]. Moreover, mTOR inhibitors block the 4E-BP1-protein phosphorylation and significantly reduce the level of E7 protein on in vitro models, leading to an accumulation of cells on G1 phase and thereby inducing apoptosis [39]. In addition, it is established that radiation activates the PI3K/AKT pathway and mTOR inhibitors sensitize tumor and endothelial cells to cisplatin and radiotherapy effects [40].

Evaluating locally advanced CC, de Melo et al. [41] recently published the results of a phase I trial combining everolimus to the standard treatment. In a 3 + 3 design the trial aimed to treat 3 dose levels of at least 3 patients with orally daily doses of everolimus (2.5, 5, and 10 mg/day), cisplatin, and radiotherapy delivered in a 9-week interval in CC patients, stages IIB, IIIA, or IIIB. Patients received everolimus from day 7 up to the last day of brachytherapy. Primary objective was to evaluate safety, toxicity, and the maximum tolerated dose (MTD) of everolimus in association with cisplatin and radiotherapy. Pharmacokinetic (PK) parameters and response rates were analyzed as secondary objectives. Thirteen patients were enrolled, 6 at 2.5 mg, 3 at 5 mg, and 4 at 10 mg of everolimus. Four patients did not complete the planned schedule, 1 at 2.5 mg presented grade 4 acute renal failure interpreted as dose limiting toxicity (DLT) and 3 at 10 mg: 1 with disease progression and 2 with DLTs, 1 grade 3 rash and 1 grade 4 neutropenia. PK results were characterized by dose-dependent increases in AUC and Cmax. Response assessment was done 12 weeks after the end of treatment and 12 patients were evaluable for response. Eleven out of 12 evaluable patients (91.6%) experienced CR and 1 (8.4%) experienced PR, with ORR of 100% at the end of treatment according to RECIST 1.1. Using the metabolic response assessment (PET/CT), 9 (75%) patients had CR and 3 (25%) had PR.

In a different scenario of patients with metastatic, persistent, or recurrent disease, a nonrandomized phase 1 clinical trial [42] evaluated 74 patients with gynecologic and breast malignancies treated with liposomal doxorubicin, bevacizumab, and temsirolimus. Thirteen CC patients were included and 10 had squamous cell carcinoma. Primary endpoints were to establish the MTD and characterize DLTs with a preliminary assessment of antitumor efficacy as secondary endpoint. All 74 patients included were heavily pretreated with a median of 4 previous chemotherapy lines. Two PR in the group of CC patients with squamous cell carcinoma (treated with dose level 6: bevacizumab 15 mg/kg IV day 1, liposomal doxorubicin 30 mg/m^2^ IV day 1, and temsirolimus 25 mg IV days 1, 8, and 15) were detected. The MTD for the study was reached at level 6. The recommended dose (RD) for phase 2 trial was bevacizumab of 15 mg/kg at day 1, liposomal doxorubicin of 20–30 mg/m^2^ at day 1, and temsirolimus of 25 mg IV at days 1, 8, and 15. The ORR in this heavily pretreated population was 20.3%. All 74 (100%) patients experienced at least 1 adverse event, mostly reversible grade 1 or grade 2, possibly drug related. Treatment combination was relatively safe and well tolerated. Among the 15 responders (CR + PR),* PIK3CA* and* PTEN* status were known in 9 (60%) and 5 (33.3%), respectively. Four (44.4%) of the 9 responders for whom* PI3KCA* mutational status was known were positive and 3 (60%) of the 5 responders for whom* PTEN* status was known were found to have* PTEN* loss.

Piha-Paul et al. [43] evaluated in a phase 1 trial 41 patients with advanced gynecologic malignancies treated with bevacizumab and temsirolimus. Six patients with CC were included and 4 had squamous cell carcinoma. Primary endpoints were to establish the MTD and characterize DLTs; a preliminary assessment of antitumor efficacy was the secondary endpoint. All 41 patients were heavily pretreated with a median number of 4 previous lines of chemotherapy. Among all patients included, 20% had SD lasting more than 6 months. Analysis of mutational status of* PTEN, PI3K, RAS, *and* RAF *was not performed in all included patients. In 2 patients who have PRs, the mutational status was not done in one and the other patient has negative status mutations for* PI3K*,* RAS, *and* RAF*. The five responders for whom* PTEN* status was known were found to have* PTEN* loss. The highest dose escalation was obtained (dose level 13: bevacizumab of 15 mg/kg IV at day 1 and temsirolimus of 25 mg/kg IV at days 1, 8, and 15) and MTD was not reached. All 41 patients experienced at least one adverse event that was possibly drug related. These events were mostly grade 1 or grade 2 and reversible; 71% of the patients experienced no treatment-related toxicity greater than grade 2.

In a nonrandomized phase 2 clinical trial Tinker et al. [44] evaluated 38 patients with CC. Primary endpoint was the objective RR as determined by RECIST version 1.1. Up to one prior line chemotherapy for metastatic or recurrent disease was allowed. Patients were treated with temsirolimus of 25 mg IV weekly in 4-week cycles. Only one patient with cervical adenocarcinoma had a PR. The median duration of SD was 6.5 months (range 2.4–12.0), 28% lasting 6 months or more (95% CI: 14–43%). The median PFS was 3.52 months (95% CI: 1.81–4.7). Eleven serious adverse events among 7 patients that were possibly related to the protocol therapy were observed. No significant association was found with any of the markers tested and response or progression on temsirolimus therapy

Due to the results of these 3 studies in a heavily pretreated population the effectiveness of therapy is not clear, and there is a tendency of activity using mTOR inhibitor treatment, but more clinical trials are needed.

## 4. Ovarian Cancer

Ovarian cancer (OC) is the most important cause of death among gynecologic cancers and, in women, it is the sixth most common cancer worldwide [45–47]. Only in the United States, 22,280 new cases of OC and 14,240 deaths were estimated for 2016 [48]. In Europe, 65,538 new cases of OC were expected for 2014 and 42,716 women were estimated to die due to this disease [49, 50]. Unfortunately, more than 60% of women with OC are diagnosed in advanced stages. The first-line treatment for patients diagnosed with OC is optimal cytoreductive surgery followed by a combination of chemotherapy with platinum and paclitaxel: this is the basis and the first choice for OC treatment [51]. The response to platinum-based chemotherapy is excellent upfront, even in high-grade serous ovarian cancer (HGSOC) [52–54], but approximately 25% of these patients acquire de novo resistance during primary treatment or relapsing within 6 months (more than 50% of the responders will have recurrence of disease) [52, 53, 55].

Patients who have PD during platinum-based chemotherapy (platinum-refractory) or with a progression within 6 months after completion of platinum-based chemotherapy (platinum-resistant) have a poor prognosis even with standard second-line therapies [49, 56–58]. Although the inclusion of bevacizumab to the chemotherapy could improve PFS for patients with platinum-resistant OC [59] there is still an urgent need to develop novel treatments based on the distinct biological background of this disease [49, 53].

Nowadays, due to a wide range of molecular profiling studies, that is, the genomic analyses conducted by the Cancer Genome Atlas (TCGA) network, the knowledge and comprehension of the molecular pathogenesis of OC have improved [51, 53, 60]. It is unquestionable that OC is an extremely heterogeneous disease with important differences not only in molecular profile and histology but also in prognosis and chemosensitivity, depending on the subtype [51, 61]. HGSOC, the most common subtype of OC, has as important characteristic, genomic instability, as well as sensitivity to platinum-based chemotherapy. Apart from* P53* mutations, which are present in 98% of cases, gene mutations that are frequently identified in other solid tumors (i.e.,* PIK3CA*) are not usually found in HGSOC [51, 62].

Clear cell, endometrioid, low-grade serous, transitional, and mucinous subtypes are examples of uncommon subtypes of epithelial OC. Different from HGSOC, these rare subtypes show, very frequently, oncogenic mutations (which could be target of novel therapies), besides genomic stability. They have better prognosis when compared to HGSOC due to the fact that their diagnosis is often done in initial stages, but at the same time, chemoresistance is a recognized characteristic of the advanced and recurrent disease [51, 63].

Oftentimes, the PI3K/AKT/mTOR pathway is deregulated in OC and* PIK3CA *mutations have been reported in approximately 12% of OCs [64–66]. The mTOR pathway is activated in approximately 70% (activated in about 50% of the HGSOC patients) [66, 67]. It is interesting that the type of PI3K alteration seems to be related to the histology.* PTEN* loss has been identified in 5% of cases of HGSOC and amplifications in* PIK3CA* in 20% and in one of the AKT isoforms (AKT1, AKT2, and AKT3) in 10%–15% of cases [51, 67].

The levels of expression of phosphorylated AKT (pAKT) and* PIK3CA* were found to be related to a lower survival rate and the activation of the pathway was found to be an independent negative prognostic marker in OC (measured by AKT or mTOR phosphorylation levels) [51, 68]. Preclinical experimental studies also suggested that concomitant mutations in the MAPK pathway (*KRAS*,* NRAS*, and* BRAF*) can mediate resistance to therapy and* PIK3CA *mutations can predict response to PI3K and/or mTOR inhibitors [65, 69, 70].

A recent paper presented in ASCO Meeting 2016 evaluated 379 patients with ovarian or fallopian tube cancer. The frequency of alterations in 10 genes (*AKT1*,* AKT2*,* AKT3*,* mTOR*,* PIK3CA*,* PIK3C2B*,* PIK3R1*,* PTEN*,* TSC1*, and* TSC2*) in the PTEN/PI3K/AKT/mTOR pathway was evaluated and determined. It was demonstrated that 33% of the OC patients have aberrations in at least one of the ten selected genes and the most common alterations were PIK3CA in 12% of the patients, followed by* PTEN* alterations in 10% and* AKT2* amplifications in 2%. Missense mutations in* mTOR*,* TSC1*,* TSC2*, and* PIK3C2B* were also identified and* TP53* missense/nonsense mutations were found in 70% of the cases [70].

Some tumor types have shown response when treated with mTOR inhibitors (temsirolimus, everolimus, and ridaforolimus) and this class of drugs has also been assessed in OC.

### 4.1. mTOR Inhibitors: Monotherapy

GOG 1701 is a phase II clinical trial that evaluated the use of temsirolimus in OC patients. Fifty-four patients were enrolled and five of them (5.3%) presented PR to monotherapy for refractory, recurrent OC, or primary peritoneal cancers [71].

In another phase II trial published in 2016, OC patients were treated with weekly temsirolimus at a flat dose of 25 mg. Objective responses were observed in 9.3% of patients and the 6-month PFS rate was 24%; hence, the study failed to meet its efficacy endpoint. However, a few patients in this study had long lasting remissions or disease stabilization under mTOR-inhibition treatment [49]. The authors concluded that the observed activity was insufficient to justify a phase III trial of temsirolimus in unselected OC patients. The trial included mainly serous tumors and only a few endometrioid (4 of 54, 7%) or clear cell ovarian tumors (3 of 54, 6%), the two subtypes most likely to demonstrate* PIK3CA* mutations. Interestingly, 1 of the 3 clear cell OCs had an objective PR to temsirolimus. More recently, one objective response lasting for 14 months and 1 SD was reported for 5 patients with clear cell OC treated with temsirolimus [72].

### 4.2. mTOR Inhibitors: Combination Therapy

Considering the limited activity of mTOR inhibitors as monotherapy and the evidence from preclinical studies indicating an additional benefit of mTOR inhibitors when associated with chemotherapy, some trials have investigated the effects of the combination of mTOR and cytotoxic drugs [51]. Phase I studies evaluating patients with gynecological tumors and treated with temsirolimus combined with weekly topotecan or pegylated liposomal doxorubicin (PLD) have been performed. However, those studies showed no expressive results as they were small and the activity was limited [19, 51, 73]. Another phase I clinical trial evaluating the use of temsirolimus plus carboplatin/paclitaxel [51, 74] showed PR in 3 of 6 patients with OC. Other two phase I studies have been reported, one combining ridaforolimus plus carboplatin and paclitaxel [51, 75] and another one associating everolimus with weekly paclitaxel [51, 76].

A phase II study evaluated a total of 140 patients with advanced breast, CC, EC, and OC when treated with mTOR inhibitors. Sixty patients (43%) had OC and P*IK3CA *mutations were detected in 12% of them. There was no significant association of* PIK3CA *mutation status with age, disease type, or ethnicity. Responses were observed only in the combination schedules, but not with monotherapy (44% versus 0%, resp., *p* = 0.06) [65].

Another phase II trial, now assessing the efficacy of temsirolimus and trabectedin in patients with recurrent clear cell carcinoma, was performed. In this study, 17 patients were enrolled and the ORR was 18% [CR = 1 patient; SD > 3 months = 5 patients (29%)] [51, 77]. Considering all the information above, it is difficult to establish final conclusions regarding the importance of the association of mTOR inhibitors and cytotoxic drugs [51]. Currently, there are ongoing trials recruiting patients evaluating the use of mTOR inhibitors (i.e., mTORC1/2 inhibitor AZD2014 or the oral AKT inhibitor AZD5363 for recurrent endometrial, NCT02208375; ovarian cancer and a phase I trial of the combination of AZD2014 and weekly paclitaxel, NCT02193633) and in view of this new data, this question might be clarified.

As previously stated, phases I and II trials in recurrence or refractory OC have shown a modest response, but the presence of confounding factors suggests that further investigation is needed [45]. Intensive research on the PI3K/AKT/mTOR pathway and its interactions with other cellular signaling mechanisms is necessary and simple solutions are not likely to be expected [49, 78].

The combination of endocrine therapy with PI3K inhibition in low-grade serous and endometrioid ovarian tumors would also be of considerable relevance as these subtypes frequently express hormone receptors [23, 45]. The association of MEK and PI3K inhibitors could also be appropriate in low-grade serous OC based on the rational that this subgroup presents frequent alterations in the RAS and PI3K pathways [51]. When proposing new therapies, it is also important to consider target specificity of the drug, that is, sirolimus, whose target is limited to the mTORC1 [51, 67].

In summary, advances in genomic characterization of OC have been seen without corresponding successful targeted therapies [51, 65]. The role of mTOR inhibitors is not completely clear or defined, especially in the context of combination therapy, which may be antagonistic or a chemoresistant promoter [51]. Histology-specific trials are important, helpful, and necessary. Further development of PI3K/AKT/mTOR inhibitors should also consider that single-agent PI3K/AKT/mTOR pathway inhibition may not always be sufficient to promote response as* PIK3CA *mutations often coexist with other concurrent molecular alterations. Moreover, predictive biomarkers in order to identify the group of patients with the highest probability to have benefit from this target therapy are still necessary [51].

## 5. Conclusion

The present study reviewed the available evidence regarding the potential impact of some mTOR pathway inhibitors in the treatment of gynecological cancer. Few advances in medical management have occurred in recent years in the treatment of advanced or recurrent gynecological malignancies, and a poor prognosis remains. Rationally designed molecularly targeted therapy is an emerging and important option in this setting; then more investigation in PI3K/AKT/mTOR pathway-targeted therapies is warranted.
